# Medical Knowledge, Religious Beliefs, and Free Will: Attitudes and Opinions of Various Undergraduate Female Respondents Regarding Oral Contraception. A Questionnaire-Based Study

**DOI:** 10.3390/ijerph18073502

**Published:** 2021-03-28

**Authors:** Bianca-Eugenia Ősz, Ruxandra Ștefănescu, Amelia Tero-Vescan, Andreea Sălcudean, Cristina-Diana Boca, George Jîtcă, Camil-Eugen Vari

**Affiliations:** 1 Department of Pharmacology and Clinical Pharmacy, Faculty of Pharmacy, George Emil Palade University of Medicine, Pharmacy, Science, and Technology of Târgu Mureş (UMPhST), 540142 Târgu Mures, Romania; bianca.osz@umfst.ro (B.-E.Ő.); boca_diana14@yahoo.com (C.-D.B.); george.jitca@umfst.ro (G.J.); camil.vari@umfst.ro (C.-E.V.); 2 Department of Pharmacognosy and Phytotherapy, Faculty of Pharmacy, George Emil Palade University of Medicine, Pharmacy, Science, and Technology of Târgu Mureş (UMPhST), 540142 Târgu Mures, Romania; 3 Department of Biochemistry, Faculty of Medicine, George Emil Palade University of Medicine, Pharmacy, Science, and Technology of Târgu Mureş (UMPhST), 540142 Târgu Mures, Romania; amelia.tero-vescan@umfst.ro; 4 Department of Ethics and Social Sciences, Faculty of Medicine, George Emil Palade University of Medicine, Pharmacy, Science, and Technology of Târgu Mureş (UMPhST), 540142 Târgu Mures, Romania; andreea.salcudean@umfst.ro

**Keywords:** oral contraceptive pill, student, religion

## Abstract

The decision to use oral contraception varies and is based on several considerations: Personal reasons, the evaluation of the benefit/ risk ratio, and religious beliefs. In this research, a questionnaire was distributed to 422 female students from the George Emil Palade University of Medicine, Pharmacy, Science, and Technology of Târgu Mureş, Romania (UMPhST Târgu Mureș, Romania), aged between 19 and 24 years old. The first endpoint of the study was to evaluate the use of hormonal contraception by the sexually active female population. The second endpoint was to assess the degree of awareness of the benefit/risk ratio of oral contraceptive use. The third endpoint was to evaluate the influence of religious beliefs regarding the decision to use this type of pharmaceutical product. Our results show that only a small percentage of students chose to use oral contraceptive pills (OCP). Fortunately, most of the respondents were well-informed and used a particular contraceptive drug based on a healthcare professional’s recommendation. Another aspect that emphasizes the choice of contraceptive method is the religious affiliation, which could influence the decision to use OCP. For the students with medical knowledge, the advice of a healthcare professional seems to be quite important, because they are aware of the risks of improper use of OCP. Although religious doctrines affect the decision to use oral contraception, this is not always taken into account, as the use of OCP is a personal decision.

## 1. Introduction

Contraception represents a set of procedures designed to prevent fertilization temporarily and reversibly. Women choose reversible contraceptive methods to prevent or postpone pregnancies for reasons related to age and/or life stages. Adolescents and young adult women often seek to postpone pregnancy until their social, educational, professional, or financial status have been secured, or such goals have been reached.

The combined hormonal contraceptives, consisting of an estrogen and a progestin, are most commonly used. They inhibit ovulation, affect cervical mucus, and alter the motility of the fallopian tubes. They also inhibit endometrial thickening making it difficult for the blastocyte to implant [1].

In Romania, OCP are issued on the basis of a medical prescription (renewable, valid for six months). In addition, there are family planning centers in most cities. Here, high school girls, students, and the female population of childbearing age can benefit from free contraceptives and specialist advice. Regarding emergency contraception, the drugs included in this category can be dispensed on request from pharmacies (they have the status of over-the-counter drugs). Abortion can be performed on request, but the pregnancy must not be older than 14 weeks; a more advanced pregnancy can be terminated if it is imposed by the health of the mother or child or is carried out for therapeutic purposes [2].

The theoretical efficiency of OCP is praised for being 100%, but studies reveal a failure rate of 0.2–1 pregnancies/100 women/year. The advantage of oral hormonal contraceptives lies in their independent use over sexual intercourse and because normal fertility is re-established once their administration stops. OCP can be used in the prophylaxis of genital and extragenital diseases as well (menstrual disorders, menstrual cramps, irregular periods, fibroids, pain related to endometriosis, menstrual migraine, or acne) [3].

Nowadays, combined OCPs are safer and more tolerable, with equal or improved effectiveness compared with early formulations. A progressive decrease in estrogen doses lowers the risk of unwanted estrogenic side effects. Concerns about the risk of venous thromboembolism are diminished with the newer generations of OCPs that have minimal side effects [4]. Nevertheless, patients with conditions associated with a risk of cardiovascular events should not use these medicines. Blood pressure levels should be assessed before initiating the treatment [5].

The most common side effects include nausea, headache, amenorrhea, and intermenstrual bleeding. Some patients experience mild nausea in the first months of treatment, but over time, the symptoms disappear. Sometimes, pills can cause breast enlargement, accompanied by painful tension. Normally, this disorder ceases after the first weeks of use. Certain hormones can trigger headaches or even migraines. The currently used pills have a lower concentration of hormones, and the risk of this side effect is diminished. In the first three months of contraceptive treatment, small hemorrhages that are not related to menstruation may occur. The problem arises because the body adapts to a different level of hormones. It is not a manifestation to worry about unless heavy bleeding persists for more than five days [6].

Data regarding weight gain are controversial [7]. Oral contraceptives can affect women’s sexual function, but sexual side effects are not well-studied, especially in terms of the impact on libido. A small percentage of women experience an increase or decrease of libido, most being unaffected [8].

Unfortunately, OCP does not protect against sexually transmitted infections. This means that choosing them as a contraception method creates the need for a second method of prevention, the use of condoms. However, less than 25% of adolescents use dual methods of contraception [9]. Therefore, counseling plays an important role for the woman to choose the best contraception method over time, thus reducing the individual risk of unplanned pregnancy and the occurrence of side effects.

In addition to issues related to their safety, the religious denomination may change the decision to take OCP or not. It weighs decisively because, depending on the religious cult, the attitude towards hormonal contraception is different. 

## 2. Materials and Methods

The study conducted in 2019 enrolled 422 students. The sample size was chosen to reflect the structure of the university’s population: Statistical data for the 2018/2019 academic year indicated a total number of undergraduate students of 9188, of which 5857 were women (63.74%). Given the number of respondents (n = 422), the sample is representative of the university’s female population, with a 95% confidence interval and a 4.60% margin of error. If we compare our sample to the general population of Romania (19,317,984 inhabitants, including 9,872,287 women, or 51.1%), we see a clear female majority; moreover, in the 19–24 age group in the Romanian population, men predominate (3.20% against 3.05%), although there is a slight female majority in the whole of the population [10].

Students were asked to complete an anonymous questionnaire. The study was approved by the Ethics Committee of Scientific Research UMPhST Târgu Mureș (Approval no. 106 of 12/04/2019). All participants were informed about the purpose of the study.

The first conference of the week for each specialty/year of study has been identified. After the investigators presented the study objectives to the class, the questionnaires were randomly distributed to 20 female students in the room. If a student refused to participate in the study, the questionnaire was distributed to another person. If this was not possible, less than 20 respondents were included for that year of study. Each student completed the questionnaire anonymously, respecting the confidentiality of the response. The interviewers who distributed the questionnaires were unrelated to the subjects included in the study (did not have a teacher-student relationship or any other type of relationship resulting in a respondent being characterized as a vulnerable subject).

Student enrolment was made randomly, as represented in Table 1.

Using a questionnaire consisting of a series of eight questions (and six additional questions in the case of students who stated that they used OCP), we wanted to assess the following: The percentage of students from different faculties who use OCP, how students chose to use a particular type of pharmaceutical product (self-medication or following the advice of a healthcare professional), how the use of OCP was influenced by the type of faculty attended (students who enrolled in undergraduate medical courses benefiting from more information), the year of study (certain courses provide the students with topics and information related to these drugs), and by religious beliefs.

## 3. Results

Our survey asked whether participants used oral contraceptive pills, and whether they knew the types of oral contraceptives? Most respondents (87.5%) stated that they did not use OCP, but had knowledge of them (64.2%).

### 3.1. The Influence of Religious Belongingness on the Decision to Use OCP

Although UMPhST Târgu Mureş is a multicultural university, 81.5% of the respondents were Orthodox, the most common religion in Romania. The distribution of the other religions was: Roman Catholic 7.8%, Reformed 5.4%, Adventist 1.7%, Protestant 0.7%, Unitarian 0.7%, Christian according to the Gospel 0.5%, Evangelical 0.3%, Greek Catholic 0.3%, Neo-Protestant 0.3%, Pentecostal 0.3%, while 0.5% declared themselves as atheists.

The confessional structure of the respondents is representative of the religious affiliation of the Romanian population, and the sample size ensures reliable data for the chosen topic. The religious affiliation (expressed as a percentage) was similar to the data published by the Romanian government in 2018. According to the State Secretary for Cults, on the Romanian territory, the religious affiliation of the population is: Orthodox 86.5%, Roman Catholic 4.7%, Reformed 3.2%, Adventist 0.5%, Unitarian 0.3%, Christian according to the Gospel 0.23%, Evangelical 0.05%, Greek Catholic 0.8%, Pentecostal 1.02%, and Atheism 0.11% [11].

Although few students belonged to denominations other than Orthodox, the influence of religious doctrines on the decision to use contraception could be observed, 16.2% of Orthodox, 15% of Reformed, and 16.7% of Adventists admitting using oral contraceptives. All students belonging to the Catholic, Christian according to the Gospel, Evangelical, Greek Catholic, Neo-Protestant, Protestant, Pentecostal, and Unitarian denominations stated that they did not use such medicines. 

### 3.2. The Influence of Specialized Knowledge on the Decision to Use OCP

An association between the level of knowledge of the safety profile of OCP and the year of study had also been identified. In the case of medical specializations (medicine, pharmacy, and dentistry), a decrease in the number of negative answers to the question “So far, have you received information during any lectures about the safety of using these products?” was observed. We also wished to identify which courses provide information about the benefit/ risk ratio of OCP, as presented in Table 2.

It should be noted that the structure of teaching programs is different between university specialties. Thus, pharmacology is studied only in medical schools, from the third year onwards in the faculty of pharmacy and general medicine, and in the second year in the faculty of dentistry. Endocrinology is studied only in the faculties of general medicine and dentistry in the 4th and 5th years, respectively, and gynecology only in general medicine in 6th year (there is evidence of high inter- and intra-variability, both for the same specialization and for different curricular programs). Moreover, some law and engineering students stated that they had information from high school courses.

The attitude towards the use of OCP was influenced after attending the courses in which the benefits and risks of using these products were presented, 60.1% affirmatively answering the question—“Has your attitude towards oral contraception changed after attending courses in which the benefits and the risks of OCP were presented?”

Students who stated that they used OCP were asked to answer six additional questions to assess:

1. The preference for a certain type of oral contraceptive pills (Q1: “What type of oral contraceptives do you use depending on the dose of ethinyloestradiol in the composition?”; Q2: “What type of oral contraceptives do you use depending on the ratio of ethinyloestradiol/progestin? A. high-dose (50 µg or more), B. moderate-dose (30–35 µg), C. low-dose (15–20 µg) of estrogen”).
2. If they used such products on their initiative or on the recommendation of a doctor or pharmacist (Q3: “Have you consulted a doctor before using oral contraceptives?”; Q4: “Do you take oral contraceptives on the recommendation of: A. General practitioner, B. The gynecologist, C. The endocrinologist, D. Pharmacist, E. On my initiative”);
3. If they had read the package leaflet before use and knew what to do when they missed a dose. (Q5: “Do you read the package leaflet before use? A. Yes, B. No, C. Not always.”; Q6: “Do you know what to do if you fail to take one or more doses? A. Yes, B. No, C. I am not sure I have the right information”).

Although there were no significant differences in the type of oral contraceptive used, depending on the dose of ethinyloestradiol (regular dose pills—32%, low dose—34%, ultra-low dose—34%), a preference was observed for the low dosed or ultra-low dosed ones in the case of senior students.

The option regarding the ethinyloestradiol/progestin ratio was interesting, most opting for monophasic (66%) and biphasic (32%) preparations. However, the students of the Faculty of Pharmacy, and especially those from the final years (4 and 5), preferred the use of low dosed, biphasic ones. Preference for the type of oral contraceptive pill is presented in Table 3.

The decision to use contraceptives, according to the respondents, was taken after medical advice (94.3%), only 5.7% (from the Faculties of Dentistry and Engineering) using these products on their initiative. In 84.9% of cases, the recommendation was made by the gynecologist, in 11.3% of cases by the endocrinologist, and 1.9% took the products on the pharmacist’s recommendation. No one consulted the general practitioner.

When asked, “Do you read the package leaflet before use?”—79.2% answered “yes”, and 20.8% said, “not always”. It could be observed that the students who attended faculties of medical sciences were more interested in the information provided in the package leaflet than those of other specializations. 

To see how well-informed they were, they were asked: “Do you know what to do if you miss a dose?”. Most, 88.7% answered “yes”, 1.9% “no”, and 9.4% “not sure”.

## 4. Discussion

The study results suggest that, although most students of UMPhST had information about OCP, only a small percentage (12.5%) used them. The selection of a certain type of OCP was based on a specialist’s recommendation, especially a gynecologist, a fact which indicates that students were well-informed and receptive to specialized advice. The study shows that none of the students followed the recommendations of the general practitioner, although these should also play an important role in contraceptive counseling. 

Whether or not they used OCP, the attitudes of the students differed significantly depending on the year of study, as in the final years of study, more information about their benefits and risks was presented, especially in the case of students attending medical specialties.

For many people, religious teachings play an important role in deciding whether or not to use contraception. The perception of different religions regarding contraception varies a lot. Even the strictest religious denominations accept contraception in certain situations. These exceptions are based on moral principles, such as responsible choice and human rights. It is admitted that family planning strengthens the family, protects the health of women and children, and prevents unwanted pregnancies. This practice was condemned by Christianity until the 20th century and was considered an obstacle in fulfilling God’s desire to procreate. The first cult to accept contraception was the Protestant one (comprising the Adventist, Baptist, Anabaptist, Anglican, Reformed, Methodist, and Pentecostal churches), considering that morality was more about each person’s conscience and less about religious teachings. However, conservative Protestants (e.g., Evangelicals) only accept these methods in the case of couples who already have children.

Moreover, all the churches included in this cult consider abortion a sin; therefore, they accept those methods that prevent fertilization, with abortion only being accepted if the mother’s life is endangered or when the fetus is not viable. The less conservative churches accept the termination of pregnancy under certain conditions and consider that the use of contraceptive methods is a moral decision of the mother. From a biological point of view, life begins at the time of fertilization, with the zygote taking over the genetic inheritance of the mother and the father, but being different. The idea is similar to that found in the Psalms of David (Psalms, 138: 13): *“For thou hast possessed my reins: thou hast protected me from my mother’s womb”*.

Contraception is still controversial in the Evangelical church. In some organized groups of people who have the same doctrine within a religion, a firm position is observed against any contraceptive method; in others, only natural family planning is accepted or the use of contraceptive methods that prevent fertilization.

The official position of the Romanian Orthodox Church has been expressed very clearly; thus, “abortion and contraceptive or abortion treatments are serious sins that kill human beings, hinder the natural procreation process of human beings, affect and endanger the dignity and lives of young women and ultimately exclude us from the Kingdom of God to which Christians aspire” [12]; however, it is stated that “if the mother’s life is really endangered by pregnancy or childbirth, priority should be given to the woman’s life, not because her life has a greater value in itself, but because of the relationships and responsibilities towards other people who depend on her” [12]. 

Roman Catholic teachings are the strictest when it comes to contraception. Any method is considered immoral because it prevents the procreative aspect of sexual intercourse. Since the progeny is considered alive from the moment of fertilization, any form of contraception that prevents the implantation of the embryo is considered a sin, an abortion. The only accepted method is abstinence. Both in the case of Orthodox and Catholic believers, abortion is only accepted if it is intended to save the mother’s life or when the fetus is deceased in utero.

Religious beliefs also influence legislation. In traditionally European Catholic countries (Poland, Malta, Monaco, San Marino, Andorra, Liechtenstein), the abortion law is very restrictive, and abortion is allowed only in specific situations. In Liechtenstein, abortion is legal only if the pregnancy represents a risk for the woman’s life/health or is the consequence of a sexual assault. Since 30 October 2020, Poland allows abortion only if the pregnancy results from rape or incest, when the mother’s life or health is at risk, or when the fetus is irreparably damaged. In Andorra, abortion is performed only to save the mother’s life [13].

The morality of contraception is still hotly debated in modern Orthodoxy. The Orthodox Church accepts only abstinence as a contraception method and considers that the sole purpose of sexual intercourse is procreation. However, the Eastern Orthodox Church accepts contraception in married couples, but only those methods that do not allow fertilization. Like other churches, the couple’s decision not to have children is accepted if it is justified: There is a risk of transmitting genetic diseases, the couple cannot support another child financially, or there is a maternal risk. Abortion and emergency contraception are prohibited [14,15].

Similar to our study, another study sought to elucidate religious and cultural influences that may affect the acceptance and use of various methods of contraception, including emergency contraception. After analyzing the articles published in the literature on religious teachings related to family relationships, sex, and family planning in different denominations (Christianity, Judaism, Islam, Hinduism, Buddhism, and Chinese religious traditions), it was concluded that religious beliefs influence the acceptance and use of contraception in different ways. Some sects interpret religious teachings related to this subject differently and are against the use of contraceptive methods. However, some women and their partners choose to ignore these teachings [14].

Medical literature reports results of studies regarding the attitude of female students towards hormonal contraception. However, their number is limited, and they usually follow emergency contraception.

A study assessed sexual activity, unwanted pregnancies, and emergency contraceptive decisions in Rwandan higher education students. During the academic year 2013-2014, 296 students aged between 18 and 25, enrolled at the University of Rwanda, were randomly recruited to complete a questionnaire. The number of sexually active girls varied between 29% and 49%, most of them having a positive attitude towards emergency contraception (67%). However, less than half had adequate knowledge (47.64%), and only 5.4% used emergency contraception [16].

Another study enrolled students from Punjab University, Chandigarh. Of the 1017 students included in the study, only 507 (49.9%) knew different contraception methods. Most knowledge was related to oral contraception (47.1%), and only 7.3% knew about emergency contraception. Of these, only 10 knew the correct timing for administering the pills, and 48 respondents were aware of their side effects. At the end of the study, the need to implement special sexual education programs was highlighted [17].

Abortion is a major public health problem in low and middle-income countries. Young and unmarried women are a high-risk group for unsafe abortions. In this regard, a study was conducted to assess the knowledge, attitudes, and experiences regarding emergency contraceptive use among the students from the University of Buea (Cameroon). The study was conducted to develop and refine the National health program and to reduce unwanted pregnancies. The study was based on interviewing 664 students. nowledge of the general characteristics of emergency contraceptive pills was low, and the degree of misinformation was alarmingly high. Knowledge differed depending on the source of information, with medical sources providing the best and most accurate information. Although, *grosso modo*, students had positive attitudes about emergency contraceptive pills, up to 65.0% thought they were unsafe. Those with adequate knowledge generally showed favorable attitudes towards emergency contraception [18].

One of the limitations of the study was that participants who used contraceptives on an endocrinologist’s recommendation were not asked whether their administration was to treat an endocrinological condition or for contraception. A weakness of the study design is that neither sexual activity nor the relationship status was taken into account, both being factors that weigh heavily in the decision to use OPC.

## 5. Conclusions

An important finding of this study is that the level of knowledge about the use of oral contraceptives in UMPhST students is quite high—most of them stated that they had information on the types of oral contraceptives. The decision to use OCP or not belongs entirely to each person, and was negatively influenced, in some cases, by the year of study, due to the lack of appropriate information. Sophomores have less information about the benefit/ risk ratio of these drugs compared with those in the final years because, in some courses, information regarding the safety of using these drugs is presented. The role of counseling for this group remains particularly important, as sexual behavior between students differs over the 4, 5, or 6 years of study in terms of frequency of sexual intercourse, choice of contraception, and responsibility. It is important to note that most students included in the survey used oral contraceptives with caution and responsibility, on the advice of a specialist who could provide them with the information they need to choose the best option for them. These young women understand the need for contraception and apply effective, modern contraceptive methods, either to prevent an unwanted pregnancy or for other subjective reasons. 

Religious beliefs can have a strong impact on the decision to use contraceptive pills. The acceptance of these products among students with religious beliefs, other than Orthodox, was lower. While health care providers need to be careful not to attribute stereotypical religious, social, and cultural characteristics to women seeking contraceptive advice, they also need to recognize that different value systems can influence contraceptive decisions. A woman’s values are not always in line with the official teachings of her religion or the cultural norms reported by other members of the same culture. 

## Figures and Tables

**Table 1:** Distribution of female students who had completed the survey by faculty and year of study.

Year of Study	Faculty				
	Medicine	Dentistry	Pharmacy	Law	Engineering
1	n = 20	n = 20	n = 18	n = 14	n = 5
2	n = 20	n = 20	n = 18	n = 9	n = 13
3	n = 20	n = 20	n = 20	n = 18	n = 14
4	n = 20	n = 18	n = 17	n = 3	n = 18
5	n = 20	n = 19	n = 20	-	-
6	n = 18	n = 20	-	-	-

**Table 2:** Distribution of information on the benefit/risk ratio of oral contraceptive treatment by courses (Answers to the question: “Please indicate which courses have provided you information about oral contraceptive pills”).

Faculty/Year of Study	I	II	III	IV	V	VI
Medicine *						
None	95.0%	100%	47.4%	0%	6.3%	0%
Pharmacology	0%	0%	53.6%	54.2%	40.6%	53.6%
Gynaecology	5.0%	0%	0%	0%	3.2%	14.3%
Endocrinology	0%	0%	0%	46.8%	49.9%	32.1%
Dentistry *						
None	100%	75.0%	66.7%	57.8%	26.3%	19.0%
Pharmacology	0%	20.0%	33.3%	31.6%	31.6%	42.9%
Gynaecology	0%	5.0%	0%	5.3%	5.3%	23.8%
Endocrinology	0%	0%	0%	5.3%	36.8%	14.3%
Pharmacy *						
None	100%	100%	83.3%	93.8%	0%	
Pharmacology	0%	0%	16.7%	6.2%	100%	
Law *						
None	100%	100%	78.0%	100%		
Engineering *						
None	100%	54.0%	43.0%	100%		

* The main courses recognized as providers of information were pharmacology (55.4%), endocrinology (26.4%), and gynecology (10.9%).

**Table 3:** Preference for the type of oral contraceptive pill correlated with the specialty.

Faculty	n	Depending on the Dose of Ethinyloestradiol			Depending on the Ethinyloestradiol/Progestin Ratio		
		High-Dose (50 µg or More)	Moderate-Dose (30–35 µg)	Low-Dose (15–20 µg)	Monophasic	Biphasic	Triphasic
Medicine	14	42.9%	35.7%	21.4%	50.0%	42.9%	7.1%
Dentistry	14	42.9%	14.2%	42.9%	78.6%	21.4%	0%
Pharmacy	10	10.0%	40.0%	50.0%	20.0%	80.0%	0%
Law	2	50.0%	0%	50.0%	100%	0%	0%
Engineering	13	23.1%	53.8%	23.1%	100%	0%	0%

A preference for high-dose monophasic pharmaceutical formulations was noted except for the case of pharmacy students who preferred the low-dose, biphasic formulations (the response could be influenced by the lack of information).
